# Living Under Coronavirus and Injecting Drugs in Bristol (LUCID-B): A qualitative study of experiences of COVID-19 among people who inject drugs

**DOI:** 10.1016/j.drugpo.2021.103391

**Published:** 2021-12

**Authors:** Joanna M. Kesten, Adam Holland, Myles-Jay Linton, Hannah Family, Jenny Scott, Jeremy Horwood, Matthew Hickman, Maggie Telfer, Rachel Ayres, Deborah Hussey, Jack Wilkinson, Lindsey A. Hines

**Affiliations:** a NIHR Health Protection Research Unit in Behavioural Science and Evaluation, Bristol, Medical School, University of Bristol, Oakfield Grove, Clifton, Bristol BS8 2BN, United Kingdom; b NIHR Applied Research Collaboration (ARC) West, 9th Floor, Whitefriars, Lewins Mead, Bristol BS1 2NT, United Kingdom; c Population Health Sciences, Bristol Medical School, University of Bristol, Oakfield Grove, Clifton, Bristol BS8 2BN, United Kingdom; d School of Education, 35 Berkeley Square, Bristol BS8 1JA, United Kingdom; e Centre for Academic Mental Health, Bristol Medical School, University of Bristol, Oakfield House, Oakfield Grove, Bristol BS8 2BN, United Kingdom; f Dept Pharmacy & Pharmacology. University of Bath, Claverton Down, Bath BA2 7AY, United Kingdom; g Bristol Drugs Project, 11 Brunswick Square, Bristol BS2 8PE, United Kingdom; h Integrative Epidemiology Unit, Bristol Medical School, University of Bristol, Oakfield House, Oakfield Grove, Bristol BS8 2BN, United Kingdom

**Keywords:** COVID-19, Drug users, Harm reduction, Injecting, Opioid Substitution Therapy, PWID

## Abstract

**Background:**

People who inject drugs (PWID) are a high-risk group for COVID-19 transmission and serious health consequences. Restrictions imposed in the UK in response to the pandemic led to rapid health and housing service alterations. We aimed to examine PWID experiences of: 1) challenges relating to the COVID-19 public health measures; 2) changes to opioid substitution therapy (OST) and harm reduction services; and 3) perceived effects of COVID-19 on drug use patterns and risk behaviour.

**Methods:**

Telephone semi-structured interviews were conducted with 28 PWID in Bristol, Southwest of England. Analysis followed a reflexive thematic analysis.

**Results:**

Concern about COVID-19 and adherence to public health guidance varied. Efforts made by services to continue providing support during the pandemic were appreciated and some changes were preferred, such as less frequent OST collection, relaxation of supervised consumption and needle and syringe programmes (NSP) home delivery. However, remote forms of contact were highlighted as less beneficial and more difficult to engage with than in-person contact. Public health guidance advising people to ‘stay home’ led to increased isolation, boredom, and time to ruminate which impacted negatively on mental health. Lockdown restrictions directly impacted on sources of income and routine. Changes in drug use were explained as a consequence of isolation and fewer interactions with peers, problems accessing drugs, reduced drug purity and reduced financial resources.

**Conclusion:**

This study captures the significant impacts and challenges of the COVID-19 pandemic on the lives of PWID. While rapid adaptations to service delivery to help mitigate the risks of COVID-19 were appreciated and some changes such as relaxation of supervised daily OST consumption were viewed positively, barriers to access need further attention. Going forwards there may be opportunities to harness the positive aspects of some changes to services.

## Introduction

In the United Kingdom (UK) and worldwide, COVID-19 has resulted in rapid and unprecedented changes to society, with public health measures to limit the spread of infection affecting housing services, healthcare systems, harm reduction and drug treatment provision (Wisse et al., 2021). These changes are expected to have severe direct and indirect impacts on People Who Inject Drugs (PWID) (Jacka et al., 2020), a population often living unstable lives in a system of fragile state-provided support.

PWID are considered at high risk of COVID-19 infection and more severe health outcomes due to their susceptibility to infectious diseases, and cardiac and respiratory diseases (Benzano et al., 2021; Jacka et al., 2020). Additional risks to PWID can be identified from a recent review of previous “Big Events” (including natural disasters and heroin shortages), which found these caused major disruptions to drug markets, negatively impacted participants’ mental health, and resultant economic instability affected the funding and coverage of harm reduction and treatment services (Zolopa et al., 2021). COVID-19 public health measures restricting social interactions to limit the spread of the virus (Baker et al., 2021) are also anticipated to pose significant challenges for PWID (Jacka et al., 2020). For instance, purchasing and consuming drugs represents a barrier to following public health guidance on social distancing (Jacka et al., 2020; Vasylyeva et al., 2020), increasing risk of virus exposure and transmission. Border closures and restrictions of international movement leading to changes in drug supply may result in changes in the drugs used (Chiappini et al., 2020; Vasylyeva et al., 2020). PWID are already high-risk for mortality due to overdose (Bursztynsky, 2019), and rises in opioid overdose in San Francisco (US) have been observed after social distancing was introduced (Rodda et al., 2020). Existing mental health and addiction issues may also be amplified in this population due to the pandemic (Zvolensky et al., 2020).

These circumstances increase the need for harm reduction and drug treatment, at a time when access to services may be reduced (UNAIDS, 2020; Wisse et al., 2021). Needle and syringe programmes (NSP) reduce transmission of blood-borne viruses, and opioid substitution therapy (OST) improves health outcomes (Platt et al., 2017). A recent review of suitable harm reduction interventions for PWID during circumstances such as global pandemics identified social distancing at NSP, and a flexible approach to OST are required to maintain harm reduction, alongside integration between harm reduction providers and providers of housing and healthcare (Wilkinson et al., 2020). UK government guidance was developed for drug treatment services (Department of Health and Social Care, 2021), and services were advised to transfer most patients from daily supervised consumption to take home doses of OST and to lengthen prescriptions, as well as to maximise interventions to mitigate risk e.g. provision of take-home naloxone. Recently, a need for researchers to collect data on patient experiences during these unique changes to treatment has been highlighted (Frank, 2021).

The LUCID-B (Living Under Coronavirus and Injecting Drugs in Bristol) study aimed to rapidly understand how the pandemic, public health measures and associated changes to services were experienced to inform future emergency response. We specifically sought to explore how PWID experienced:

1) Challenges relating to the COVID-19 public health measures;
2) Changes to OST and harm reduction services;
3) Perceived effects of COVID-19 and service delivery changes on drug use patterns and risk behaviour.

## Methods

LUCID-B was a rapid qualitative semi-structured telephone interview study.

### Setting

The study was conducted in Bristol, which has the highest age-standardised mortality rate for deaths related to drug use in the Southwest of England (7.6 per 100,000 in 2017-19) (Office for National Statistics, 2020). In Bristol, drug and alcohol services are delivered by Bristol ROADS (Recovery Orientated Alcohol and Drugs service), a partnership between Bristol Drugs Project, Developing Health and Independence (DHI) and Avon and Wiltshire Mental Health Partnership (AWP) NHS Trust. BDP are an independent agency offering a range of services for people who use drugs and alcohol including: BDP and pharmacy delivered NSPs; shared care OST prescribing with GPs (care jointly provided by GP and BDP, with GPs taking responsibility for prescribing and monitoring, and BDP providing regular psychosocial input and support through a shared care worker); a nurse led service for drug-related physical health issues; and mutual aid groups (see www.bdp.org.uk for further information). Other services used by PWID in the city include a Homeless Health Service offering primary care and OST prescribing; temporary accommodation including drug treatment hostels; an inpatient detoxification unit; and mutual aid groups provided by DHI and other charitable organisations.

Data were collected from 11^th^ June – 4^th^ August 2020. The first UK national lockdown was from 23^rd^ March – 4^th^ July; initially, legislation prohibited the public from leaving home without a reasonable reason and closed all but essential businesses; from early June groups of up to six could meet outside, retail businesses opened, and requirements to wear face coverings began to be introduced; by August, two households could meet indoors, and pubs and restaurants opened (Baker et al., 2021). Throughout this period, government guidance recommended social distancing measures (Cabinet Office, 2021). On 17^th^ March government funding for local authorities was announced for the ‘Everyone In’ scheme, which led to the rapid housing of people who were street homeless in commercial accommodation including hotels, to enable them to self-isolate and prevent COVID-19 transmission (Ministry of Housing, Communities and Local Government, 2020).

Services for PWID in Bristol had to adapt in response to these public health measures, mostly resulting in changes to form or route of delivery rather than function (Table 1). Essential face-to-face services (pharmacies and healthcare) continued with social distancing measures in place, but as far as possible interactions between service providers and service users (shared care OST and healthcare appointments) were conducted remotely predominantly by telephone. Some mutual aid groups stopped, with others delivered online or by telephone; the inpatient detoxification unit closed; and some pharmacy NSP services were withdrawn. Efforts were made to commence as many PWID as possible on OST, requirements for supervised consumption of OST were relaxed and service users were provided with up to two-weeks take-home supply.

**Table 1:** Service delivery changes under COVID-19.

Service type	Pre-pandemic	Service changes
**General service provision**	• Most services delivered face to face.	• Services provided remotely by telephone and online. • Access to some services affected by reduced opening times (at least initially), closure (residential rehabilitation, some groups and inpatient detoxification), initial lack of awareness of changes, lack of communication devices (no phone or internet access) and reluctance to discuss issues remotely or in less private spaces.
**Needle and syringe programmes (NSP)**	• Fixed site BDP NSP. • Fixed site community pharmacy NSPs. • Mobile harm reduction van serving areas on outskirts of Bristol.	• BDP NSP delivered as doorstep service. BDP NSP doorstep service (people could attend BDP and wait outside, and a BDP team member would supply an NSP kit). • BDP home delivery NSP service made widely available. • Some community pharmacy NSPs closed, others had restricted opening times, problems with equipment supplies and long waiting times. • Scaled up outreach service offering NSP in public locations and temporary accommodation including commercial providers in the Everyone In scheme.
**Opioid Substitution Therapy**	• Prescribed by GP and BDP shared care service and Homeless Health Service. • Face to face appointments to commence/renew prescriptions and provide psychosocial support. • Collected from community pharmacies.	• Rapid scripting service created. • Most shared care appointments over the telephone. • Some have requirement for supervised consumption relaxed and longer prescriptions issued with reduced frequency of medication collection. • BDP deliver prescriptions to clinically vulnerable. • Long waiting times in pharmacy
**Other drug treatment services**	• Multiple mutual aid groups provided by BDP and other organisations. • Residential rehabilitation services. • In patient detoxification unit.	• Mutual aid groups initially suspended, with some delivered online or via telephone. • Rehabilitation services closed to new clients. • In patient detoxification unit closed.
**Health services**	• Primary care services provided by GP practices and Homeless Health Service. • BDP nurse-led physical healthcare service for people who use drugs.	• Primary care appointments mostly conducted by telephone. • Continued provision of BDP nurse-led healthcare including hepatitis C in public spaces, particularly as part of the outreach service.
**Housing**	• Various homeless night shelters. • Temporary accommodation including drug treatment hostels.	• Homeless night shelters closed. • Street homeless temporarily housed in commercial accommodation as part of Everyone In scheme. • Accommodation providers institute measures to reduce risk of transmission, with reduced face-to-face staff contact in many cases.
**Criminal justice service**	• “Activity days” part of probation services usually conducted to benefit individual on probation	• Activity days postponed.

BDP re-deployed many of their staff into assertive outreach roles. The BDP NSP operated a doorstep service from its fixed site while a home delivery NSP service was made widely available. OST was delivered to clinically vulnerable service users and an outreach service worked in public areas and at homeless accommodation delivering NSP, nursing care, hepatitis C testing and general psychosocial support.

### Patient and public involvement

Two people with lived experience of injecting drugs gave feedback on our interview topic guide, our planned recruitment procedures and other study materials to ensure our questions and study information were appropriate.

### Ethics

Ethical approval was provided by the Faculty of Health Sciences Committee for Research Ethics, University of Bristol (study ref no: 105042).

### Recruitment and sampling

Participants were required to be at least 18 years old, English speaking, and injecting drugs at the beginning of the national lockdown.

BDP staff JW and DH identified participants who used their static services or through their outreach work. The study was explained using a participant information sheet and if consent was given their contact details were shared with the research team. A range of experiences were captured by purposefully recruiting through three routes (NSP home delivery service, hotels/hostels part of the ‘Everyone In’ scheme, street outreach) and inviting people with different demographic characteristics (age, gender, housing status) and drug treatment status. For example, those living in private accommodation were anticipated to face different challenges than those offered accommodation during through the ‘Everyone In’ scheme.

Because of the pandemic, it was necessary to conduct telephone rather than face-to-face interviews to reduce the risk of COVID-19 transmission. JK and AH conducted interviews during BDP outreach sessions or at a pre-arranged time. Participants borrowed a telephone from BDP workers if necessary. The interviewer asked participants if they were in a safe place where they could talk freely and confidentially and audio recorded verbal consent was taken. Participants received £10 cash as thanks for their time.

### Interview topic guide

Topics included demographic information, general experiences during the pandemic, impacts on drug use, experience of service adaptations and responses to public health measures (see Supplementary Materials). The interview topic guide was applied flexibly and adapted to reflect the changing lockdown and data collected.

### Epistemology

The study was designed and undertaken implicitly assuming the core tenets of critical realism; that is, there is a perspective-independent reality, but it is not possible to give an account of it devoid of perspective (Maxwell, 2012). Accordingly, analysis involved reflection on both the data itself and the perspectives of the researchers, aided by regular multidisciplinary team discussions (see acknowledgements for team expertise).

This project was conducted with a multidisciplinary team. A core group of researchers (AH, HF, JK, JS, LH, MJL) - none of whom report lived experience of injecting drugs - led the analysis with support from the wider team including harm reduction experts delivering drug services (DH, JW, MT, RA).

### Data analysis

With informed consent, audio recordings were transcribed and anonymised and an in-depth reflexive thematic analysis was undertaken (Braun & Clarke, 2013). A combination of deductive coding, based on the aims of the study and inductive coding, conducted for three transcripts by JK in QSR NVivo, was applied. Each of these transcripts were then independently coded by a second researcher (AH, MJL, and LH) to ensure each code was understandable; and to identify missing codes. The purpose was to support the researchers to code the data not to assess coding agreement. After discussion and further refinement, the coding framework was applied to the rest of the transcripts by the researchers (JK, AH, HF, MJL, LH), with further codes added inductively. JK led the next stage of the process reviewing all the codes and coded data within them to identify patterns of similarity, creating ‘candidate themes’ and a visual thematic map containing interconnected overarching themes, themes and sub-themes with a ‘central organising concept’ related to the research aim and objectives. These candidate themes were discussed and revised with the wider multidisciplinary research team by assessing how well they fit with the dataset, how they relate to each other and how well they address the overall research question before being finalised (Braun & Clarke, 2013). During this process, some provisional candidate themes were combined, divided or removed because they did not provide a coherent story of the data or address the research aim (Braun & Clarke, 2013).

## Results

### Participant characteristics

Interviews lasting 20-56 minutes were conducted with 28 participants. For participant characteristics see Table 2. There was no consistent pattern of change in drug use since the start of the pandemic; participants reported a range of behaviour from increases to reductions, including two who reported becoming abstinent. Two people attributed their own or another's overdose during this period to polydrug use and fluctuations in drug supply.

**Table 2:** Interview sample characteristics

Characteristic	N
Recruitment route	
Outreach	15
NSP home delivery	9
Fixed-site drug servce	4
Partially recruited (agreed to have their contact details passed onto the research team could not be contacted)	3
Gender	
Female	9
Male	19
Age	
25-29	2
30-34	4
35-39	10
40-44	5
45-49	3
50-54	4
Housing^1^	
Street homeless	1
Temporary: Homeless sleeping pod	1
Temporary: Unspecified	1
Temporary: Hotel	2
Temporary: Hostel	10
Temporary: Bedsit	1
Temporary: Drug treatment hostel	4
Council housing	3
Living with family (parent)	2
Private tenancy	3
Injecting	
Yes	26
No	1
Missing	1
Injecting years	
1-4	6
5-9	3
10-14	3
15-19	3
20-24	7
25-29	3
30-34	1
35-39	1
Missing	1
Drugs currently / recently used^2^ (N.B. participants reported a range of polydrug combinations)	
Alcohol	7
Alprazolam	1
Amphetamine	1
Cannabis	6
Cocaine (powder)	2
Diazepam	1
Gabapentin	1
Heroin (without crack cocaine)	7
Heroin and crack cocaine	21
Ketamine	1
Pregabalin	1
Spice	5
OST	
Yes	23
No	5
OST type	
Methadone	19
Buprenorphine	4
Reported changes to frequency, methods and types of drug use^3^	
Drug administration change (e.g. smoking more/less, injecting more/less,	4
Increased frequency	8
Reduced frequency	8
Variable frequency	3
Stopped use	2
No changes	1
Change of drug	3
**Total**	**28**

Findings are presented in three themes with supporting subthemes, illustrated with anonymised verbatim quotes. The ‘Attitudes to COVID-19’ theme captures concerns about COVID-19 and adherence to public health measures. The ‘Sense of Support’ theme focuses on how people felt their needs were met through their interactions with rapidly adapted services. These ‘topic summary’ themes provide important contextual information for the final theme (Braun & Clarke, 2020): ‘Sense of Loss’. This theme explores how for many, the pandemic exacerbated existing difficulties, highlighting absent things which could otherwise have improved their lives. Considerations of how COVID-19 impacted drug use patterns and risk behaviour run throughout the themes.

### Attitudes to COVID-19

Some participants were not concerned about COVID-19 infection, with perceptions of low personal risk related to not knowing anyone who'd had it, not having many contacts with others anyway, taking measures to prevent infection and awareness of low case numbers in the Southwest of England. Others were not concerned, or were in denial, about their risks of being infected; reasons for this included beliefs about immunity from previous infections such as the flu. A fatalistic attitude of COVID-19 being unavoidable was also expressed:

*I'm not, to be honest. I don't know. No, it's quite rare when you think about it. It's only one in a thousand people get it (…) To be honest, I don't personally know anyone who's had it, so it's not really affected me that much.* Interview 17, Male

Interviewer: How concerned are you about it, about getting the virus?

*Personally, I haven't really been that concerned. I just think if you're gonna get it you're gonna get it, if you're not you're not, if you're gonna die you're gonna die, if you're not you're not.* Interview 10, Male

A backdrop of more pressing issues such as drug dependence and other health conditions explained a lack of concern about COVID-19 and difficulties adhering to public health restrictions:

*I wake up unwell every morning and I've got to see the chemist for a start (...) I was allowed to do that but then obviously (…) standing around trying to earn your money and then getting your drugs is a lot of hours to be out when you're not supposed to be out at all.* Interview 11, Female

While some had experienced symptoms of COVID-19 and a small number had self-isolated or been tested, it was noted that similar symptoms are common among people who inject drugs. Consequently, symptoms could be ascribed to other causes, explaining a lack of concern:

*When the virus first came about, I did have a bad chest but I have… and a lot of people in here [temporary accommodation] do suffer with symptoms that [laughs] do mimic Coronavirus. They cough a lot. We have got bad chests from smoking drugs and… When you're on heroin you tend to sweat, hot and cold flushes, so I wouldn't be surprised if there had been a case of it and it had gone unnoticed.* Interview 28, Female

Conversely, COVID-19 infection concern related to perceived susceptibility. For instance, one female participant explained “*I was really paranoid about it because I've got bad lungs anyway”* (Interview 16). Furthermore, concerns related to exposure to peers and exposure to dealers not following public health guidance. Perceived severity of COVID-19 infection, awareness of others infected with COVID-19 and the risk of infecting others heightened concern.

There was good awareness of the lockdown and social distancing guidelines obtained through the media and word-of-mouth. Many felt other people were not adhering to the guidance but they were doing their best to, describing increased hygiene measures, avoiding contact with others, wearing masks and gloves, and staying inside.

Some people used drugs alone more due to lockdown restrictions and concern about COVID-19, some were using more with others (due to the need to pool limited resources to buy drugs) and others did not change who they used with:

*Someone you could be using with could have that [COVID-19]. So, I was using more on my own since that was about. Maybe sounds like keeping myself safer but in the long run not ‘cause obviously if I'd gone over [overdosed] there'd be no one there.* Interview 9, Male

Those forced to “break a few rules” described instances of forgetting to follow the guidelines due to intoxication and how the perpetual cycle of earning money, buying and using drugs was a barrier to adherence. For example, public drug deals were more difficult to conduct socially distanced. While some dealers initially wore masks and gloves, others were perceived to be unphased by the pandemic and continued practices like carrying drugs in their mouths.

*When the lockdown started, it was very difficult being only allowed to go out once a day for exercise. So, I was having to use that time to obviously go and meet my dealer to get my drugs, or sometimes just hoping that I wasn't going to get stopped by the police and I'd go out and obviously if I was to be stopped to say I was exercising. It did make things very difficult.* Interview 3, Male

Participants who were concerned about contracting COVID-19 or were worried about breaking the lockdown restrictions found buying drugs more difficult. Also, some dealers were reluctant to meet due to increased visibility and concerns about COVID-19:

*A lot of the dealers obviously were scared to come out because they'd stand out like a sore thumb, everything's quiet and then they're hanging around.* Interview 3, Male

Living conditions in hostels were described as not conducive to social distancing and rules against visitors were sometimes broken:

*The place that I live and the environment that I'm in it really would be like fighting a losing battle. (…) I think it's quite impossible for the residents, yeah, definitely. With stairwells and they're all down the corridor and it's all so compact in here, yeah.* Interview 28, Female

One suggestion was made for the instalment of handwashing facilities in public spaces to support hygiene measures; especially during the early lockdown when shops and restaurants were closed.

The narratives related to concern about COVID-19 and barriers to adhering to public health measures highlight the importance of appropriate communication methods and structural level mitigation measures (such as the alterations in service provision described in Table 2) which minimise risks while not placing sole responsibility on individuals who may have significant competing issues to manage.

### Sense of Support

Participants appreciated the efforts made by services to continue providing support during the lockdown. Some service delivery adaptations were preferred and seen as improvements, while others were less well-received but were viewed as “better than nothing”. Many participants did not suggest any improvements to services, believing providers were doing as much as they could. This sense of enhanced support through the ‘Everyone In’ scheme is illustrated in the following quote:

*There's actually been bonuses to it* [COVID-19]*. Like I mean having more attention from services and stuff. That's how it's felt, like they've cared more about our welfare to a degree. So, I mean I was left sleeping under a bridge for nine months and the moment the coronavirus happened it was like ‘come on, come and get in a room’.* Interview 22, female

To some extent differences in how service adaptations were experienced, and overall experiences of the pandemic, can be attributed to an individual's pre-COVID-19 circumstances. Those with indicators of greater stability and resources before the pandemic such as housing, income, health, drug use and treatment tended to be affected less (in some ways), by the public health measures and experienced less pronounced impacts on drug use, than those with less stability. For some, the pandemic coincided with a critical point in their recovery, disrupting access to the intensive support and connection with others in recovery provided by residential detox services. For others with less stability in their lives pre-COVID-19, such as those experiencing homelessness, accounts reflect improvements in some aspects of support received although many vulnerabilities were exacerbated.

Enhanced outreach service provision ensured continued service access and even improved access, convenience and support, while also helping mitigate the risks of COVID-19.

Rapid, proactively offered OST prescribing helped overcome reduced drug access and supported self-isolation:

*Before COVID-19 it used to take a couple of weeks [to start a script] so they did come through on that front by getting as many people scripted as fast as they can [same day] so they could self-isolate if they need to.* Interview 27, Male

Less frequent OST collection and relaxation of requirements for supervised consumption in pharmacies were viewed favourably; exposing individuals to drug using peers less, reducing stigma and embarrassment when publicly collecting medications and granting greater autonomy:

*I just don't have to go to the chemist every day* [relaxed OST collection requirements]*. (…) It's less exposure. There's always addicts and people crawling around outside there. (…) It's a form of control.* Interview 13, Male

However, for one person daily OST collection was viewed positively as it represented a reason to go out:

*It's a lot easier and that, but in a way, I've been doing it for years, picking up daily, so that was kind of my exercise in a way as well, getting out.* Interview 3, Male

NSP delivered as a doorstep service (see Table 1 for description) at BDP, was viewed by some as a minimal change with little impact on willingness to have open conversations with staff, due to good pre-existing relationships.

In contrast to these forms of outreach and enhanced provision, for those using services which had to be travelled to, such as pharmacies, reduced opening times (at least initially) and social distancing limits on the number of people allowed inside resulted in large queues and long waiting times for collection of OST and injecting equipment. There were also occasions when pharmacies ran out of equipment or stopped running their NSP which led to greater equipment re-use and sharing. Some people attributed increased infections, abscesses, wounds, pain, missing veins and swelling to equipment shortages as well as increased drug use and poor drug quality/purity. Others did not perceive these issues to be any more prevalent than usual and a small number felt their health was better.

*At first, we were using like four times we were using a pin four, five times and it was hard to break through the skin because it was that blunt you know what I mean. Because we're in ((area 3.4 miles from Bristol Drugs Project city centre NSP)) and ((location of Bristol Drugs Project)) is in town. So, it was you know what I mean it was hard work. I've made a right mess of my legs hence why I need the wound kits.* Interview 2, Male

Queueing publicly outside busier than usual pharmacies to collect OST was also disliked, caused anxiety and on some occasions meant missed OST dispensing.

NSP home delivery provided by BDP (see Table 1) for those stably housed was viewed positively; it was convenient, discreet, required less effort and overcame barriers to accessing equipment in pharmacies. Increased outreach service provisions such as NSP home delivery was seen to have reduced the risk of COVID-19 by limiting congregation, supporting self-isolation and removing exposure to peers for those attempting to reduce their drug use.

*They've* [NSP staff providing home delivery of injecting equipment] *been prompt, they've been accessible, they're easy to talk to, they're non-judgemental, they try and get everything that you've ordered down.* Interview 4, Female

However, the public nature of NSP home delivery and doorstep NSP at BDP restricted willingness to have open conversations. One female participant was also concerned about her daughter becoming aware of her injecting due to the NSP home delivery:

*I want to get them [BDP NSP home delivery staff] as far away from her [daughter] as possible, I don't want her to know too much, you know? (…) They gave some foil to me, because the last time they came, I come into the house, she was downstairs and said ‘who's that’? I was just like, it's [inaudible] oh yes, that's for me, for foil. But she don't know nothing about the syringes I've got put away, they're well hidden in my bedroom.* Interview 16, Female

The ‘Everyone In’ scheme (see Table 1) was positively received, though the experience of living in the accommodation varied with noisy environments at night-time and poor food reported. While some anticipated being offered longer-term housing and welcomed support from key workers, there was widespread concern caused by not knowing what support would be offered when the scheme ends. Communication around this issue was not forthcoming and promises of support were not always realised. Indeed, one participant emphasised the scheme had not solved the issue of homelessness:

*Homelessness is still a tangible problem because the government have addressed the actual pragmatic physical problem of putting a physical roof over somebody's head. It doesn't address all the complex issues as to why that person continually ends up back in the same situation over and over again.* Interview 13, Male

For some, emergency accommodation shared with people who use drugs encouraged use.

In contrast, rules against using drugs in emergency accommodation were followed by some, meaning injecting outdoors or using less as this participant describes:

*I used to use a lot with other people and now there's not so many people around me I'm not using much. Me and my missus would never get clean, but now we're in the hostel so we can lock ourselves away and ignore the door and stay away from everyone.* Interview 25, Male

Others broke the rules and were concerned they might be evicted: *I have been using in the hotel, but I worry about doing so.* Interview 12, Male.

Drug-treatment housing continued to issue residents with warnings and punitive measures following positive drug test results despite a reduction in support for substance use (e.g. residential group work stopped):

*They've* [accommodation] *still been giving the warnings out, even though there hasn't been the support here.* Interview 19

### Sense of Loss

#### A loss of routine and income

Guidelines to ‘Stay home’ disrupted patterns of daily life and resulted in boredom which was the most common reason cited for increased drug use:

*Boredom and a lot of the time because there's nobody about; you've got the money, there's nobody to talk to, nothing to do, you're just sitting there piss bored and it's like you know what? Fuck it, I'll go and have a little use and waste a few hours, you know what I mean? And then one use is two, then three, then four and before you know it you're back there with a habit on your back.* Interview 27, Male

Although one person described their accommodation supplying activity packs, there was a desire for more activities to structure the day, especially in temporary housing:

*You get pissed off of being bored to be quite honest with you and doing nothing. A big part of my day before was going out earning and scoring and all that. Now I'm not doing that, sat about all the time watching TV when I was in the hotel and there's only so much nothing you can do. It was doing my nut in.* Interview 10, Male

The lockdown directly impacted on sources of income which negatively impacted on the ability to afford drugs. Two participants spoke of vicious cycles of being in debt to dealers. Job losses combined with no job opportunities, or being placed on the government's furlough scheme, impacted on income and daily routine. Those reliant on mendicancy described few people on the streets, with those who were around not carrying cash as shops switched to only accepting contactless payments. The public were “too scared” to put money directly into the hands of those asking for it and were reluctant to come near them due to distancing guidance, which intensified felt stigma and dehumanisation:

*They make you feel like I'm a fucking disease. They won't come near you and they just chuck things at you. Before they just placed it in the hat and probably stand and have a conversation with me but now they just want to rush on by.* Interview 23, Male

First and second-hand accounts of committing and / or being a victim of opportunistic theft, stealing from peers and reportedly increasing levels of violence stemmed from difficulties accessing money. For example, shoplifting became difficult when non-essential shops closed and, queuing was necessary for entry and customer numbers were limited in open shops:

*People's anonymity has gone because shoplifters have to queue up to shoplift now, which has made violent and nasty crime.* Interview 13, Male

*I wasn't on a script and I couldn't beg so basically, I was walking down the street and the streets were dead and I took a thing off the back of this lorry but I did in right in front of the two cameras.* Interview 25, Male

For some the inability to make money through usual means reflected a positive change in relation to reduced drug use:

*Me and my partner [who had recently found out she was pregnant] well she's reduced [drug use] more than me but we've reduced drastically you know what I mean? My life was chaos and I mean chaos, shoplifting four, five times a day, supporting a £200 habit between two like so I'm doing alright.* Interview 2, Male

Reduced financial resources and increased drug prices contributed to reduced drug use, particularly for those already motivated to stop pre-pandemic.

*As soon as the virus come about my drug use plummeted to the point that I stopped using for a little bit completely, for a couple of weeks (…). It was hard to go out shoplifting; it was hard for me to go out and make money and I decided to stop. In some senses the virus has helped me, it sort of like cut my drug use right down.* Interview 10, Male

#### A loss of connection

Lockdown measures led to isolation from friends and family (apart from co-habiting partners), and time to ruminate, all of which negatively impacted on mental health. For example, one female participant spoke of not visiting her children and parents to protect her family:

*I haven't seen my children. They live with my mum and dad over in ((city nearby)). I have got the option to see them. I can see them whenever I please but because of this virus I haven't. I've chosen not to. I don't want to put them in any danger, not my family or my children. So, in that aspect it's been heart-breaking.* Interview 28, Female

For those with poor mental health, isolation caused by the lockdown worsened symptoms:

*A lot of depression as well since this COVID-19 isolation. It ain't very good just sitting in your own mind all the time.* Interview 20, male

*For people who struggle with mental health issues and addiction just being in is the last thing you want because you end up getting less money and more times on drugs. (…) It's obviously time to stew and reflect on things but also just the actual financial side of it like I lost my job and my flat I was in.* Interview 26, Male

Others felt less affected by the isolation due to pre-COVID-19 marginalisation and seclusion

*There hasn't really [been any change] for me personally because I basically live on the fringe of society anyway so the only people I really chat to are the people that are willing to chat to me anyway so yes, it hasn't particularly made a massive difference to me.* Interview 24, Female

Enforced isolation and fewer interactions with peers combined with problems accessing drugs and reduced drug quality/purity including suspicions drugs had been adulterated (e.g. fentanyl) contributed to reduced drug use, particularly for those already motivated to stop pre-pandemic. Other responses to impacts on drug supply included pooling resources and sourcing drugs with others, stockpiling, travelling further to meet dealers and changing to more readily available and cheaper drugs including synthetic cannabinoids and one instance of changing from ketamine to heroin and crack.

*I used to do it more on my own and now obviously when it's harder to come by so if someone wants to get something you might put in together.* Interview 10, Male

The lockdown also negatively affected connection in the form of communication, initially resulting in a lack of awareness of service changes. Word-of-mouth communication between peers was more difficult. Verbal and written information sharing about service alterations were vital. Many street-homeless or vulnerably housed people did not have phones or internet access to connect with services. For these people, there were feelings of being “out there blind” and unable to access help. A scheme to provide phones run by the charity St Mungo's and the Homeless Health Service in Bristol was critical:

*They must have given out hundreds of these things, the cheap £10 phones. A lot of people didn't have a phone so they couldn't have got any access to anything without it because you can't go and speak to the person because of social distancing and if you've not got a phone or internet you're not getting to see or speak to that person.* Interview 8, Female

While for some participants switching to telephone OST appointments was easier due to reduced travelling, many accounts described greater telephone and online contact as *“just not the same”*. For example, one person expressed concern OST prescriptions would be stopped if phone calls were missed. Remote contact was less beneficial, tiring, impersonal, difficult to know if the person was listening, and difficult to openly discuss issues. Similarly, online drug support groups removed informal socialising and rituals around travel and took up less time in the day. The importance of connection in drug treatment and the disruption to this caused by the pandemic is emphasised by this participant:

*They say the opposite of addiction is connection but how are you supposed to connect with people when you're not legally allowed to do that.* Interview 8, Female

Concerningly, some perceived GP practices to be closed/inaccessible and others described less frequent appointments and more self-care:

*Where my doctors is, that's all shut. (…) It shut temporarily during the coronavirus. They're only doing telephone appointments and stuff like that, which is not the same thing. (…) I've spoken to them a couple of times (…) but I haven't actually spoken to the GP, no (…) you just get the answer phone all day.* Interview 13, Male

#### Greater visibility

Previously private and hidden activities were made visible during the lockdown. The streets being empty made scoring drugs discreetly difficult and being on the streets during this period was seen as tantamount to being *“up to no good”*:

*So, there's a lot of people walking around to try and get drugs. Like you all stand out like a sore thumb because you all look ill or rough or like you're using.* Interview 21, Female

Doorstep NSP removed privacy and anonymity; contributing to reluctance to access the service and have open conversations about drug-related issues. Concern was also expressed about arrests for drug possession due to visibility of the doorstep NSP:

*When I did walk past* [Bristol Drugs Project] *a few weeks ago and there was a queue outside (…) it's not usually a queue people would want to be seen stood in. I don't think people like that very much.* Interview 28, Female

## Discussion

This study captures PWID experiences of the COVID-19 pandemic. Concern about COVID-19 and perceptions of susceptibility and severity varied. While many participants expressed intentions to follow social distancing and lockdown measures, some found this difficult due to living conditions and a perpetual cycle of earning money, buying and using drugs. Impacts of public health measures and service delivery changes were less pronounced amongst those who possessed greater stability before the pandemic. Efforts made by services to continue providing support during the pandemic were appreciated and some changes to harm reduction services, such as less frequent OST collection, preferred. Importantly, remote forms of contact with services were unable to meet some people's needs and preferences, and barriers to access were apparent (e.g. lack of phones). Loss of routine and income and social support worsened mental health problems and boredom due to “stay at home” guidelines was cited as a reason for increasing drug use. In contrast, isolation and fewer contacts with peers contributed to reduced drug use, particularly for those motivated to stop before the pandemic.

Key challenges relating to the COVID-19 public health measures were around feelings of loss, and low perceived personal risk from the pandemic, which intersected with factors specific to drug use and mental health. Quantitative work amongst PWID suggests those with recent substance use are less likely to follow distancing measures (Genberg et al., 2021). Barriers to following public health measures in this study, including structural inequalities such as living conditions, are in line with findings that the most socially and economically disadvantaged face greater obstacles to adherence (Atchison et al., 2020; The Independent Scientific Advisory Group for Emergencies, 2020). Risk to family members was a reason to isolate from significant others; this social influence, and moral obligation towards others were found to modestly predict intention to socially distance in an Australian survey, which tested an integrated social cognition model (Hagger et al., 2020). Recent ethnographic research of populations of PWID during COVID-19 has highlighted the importance of considering the dynamics of relatedness, isolation, and solitude in experiences of both substance use and social distancing (Roe et al., 2021). In combination with existing evidence, the present findings indicate specific support may be required for PWID in maintaining connection and ensuring adherence to public health guidance.

Changes in OST provision and assertive outreach were experienced positively, but there were indications remote service provision and decreased privacy in service access may present barriers for PWID. Work from Scotland has highlighted concerns regarding unsupervised consumption, including pressures to divert medication (Schofield et al., 2021), but as in recent quantitative work from the US (Figgatt et al., 2021) there were few reports of OST diversion in the present study. In the North-West of England, COVID-19 restrictions resulted in large reductions in NSP usage and the number of needles distributed (Whitfield et al., 2020), but this was not observed in our sample. This may be due to rapid changes to assertive outreach and NSP home delivery, which were well received and cited as addressing barriers to accessing equipment in pharmacies. These changes may have reduced accessibility issues which have emerged in recent North American research (Russell et al., 2021). Research from jurisdictions where NSP home delivery was not possible identified this as a barrier to treatment access (Seaman et al., 2021). However, as in other settings where COVID-19 measures led to outdoor NSP provision (Seaman et al., 2021), there were some concerns about privacy. The move to provide drug and health service consultations remotely was not universally well-received, which reflects findings from other groups with mental health issues during COVID-19 (Gillard et al., 2020). Services may wish to consider recent innovations from providers in the US (Tringale & Subica, 2021) to overcome these issues.

The public health measures and social disruption of the COVID-19 pandemic impacted upon drug use, with instability, boredom and isolation all identified as drivers of change; however, the extent to which these changes will sustain as public health measures ease is unknown. Other UK and Canadian studies observed COVID-19 measures influenced drug use (Ali et al., 2021; Croxford et al., 2021; Public Health England, 2020; Public Health England et al., 2020; Schofield et al., 2021; Scott et al., 2020), and in the US COVID-19 related stressors were linked to relapse (Hurley et al., 2021). Consequently, the issues with supply and changes in substance use during COVID-19 appear to have affected PWID globally. Although there was limited reporting of overdose in the present study, decreases in drug use are likely to be accompanied by resultant changes in tolerance. There is a need to consider these changes in relation to the drug-risk environment, which Grebely and colleagues highlighted may be worsened by COVID-19 (e.g. neighbourhood deprivation, cost of living, lack of employment opportunities) (Grebely et al., 2020). As policy makers and service providers reflect on the potential impacts of COVID-19 adaptations, it will be important to consider how to minimise the effects of an increasingly high risk environment amongst a population which has undergone rapid shifts in their drug use behaviours.

This study highlighted the need to support PWID holistically with joined-up strategic cooperation amongst local service providers, addressing reduced income, housing access, difficulty managing mental health and support for drug use; this is in line with recent recommendations for drug policy in the UK (Black, 2021). The pandemic could be seen as a ‘window of opportunity’ to rethink policy and practice, with some impacts viewed as positive by PWID (e.g. changes to OST provision) (Wisse et al., 2021). For example, decision-making about continuing changes made to OST prescribing should be informed by evidence these changes are viewed positively by service users. More research is required to explore the effect of recent changes on treatment outcomes and mortality to inform policy decisions. If changes to remote service delivery through telecommunications are maintained, it will be important to ensure PWID are not digitally excluded.

## Limitations

Our recruitment approach means the sample may not reflect the views of those who are most disengaged from services and potentially in greatest need. However, recruitment via outreach is expected to have reached beyond those who usually access drug services – as evidence by some participants being unknown to the drug service recruiters. Beyond asking participants if they were in a safe and confidential space they were happy to talk to us in, we could not guarantee whether or not parts of the interview would be audible to anyone in the vicinity of the interviewee. Although we involved PWID in the design and set up of the study, a limitation is we did not involve PWID in the analysis or interpretation of the study findings.

## Conclusion

The COVID-19 pandemic presented significant challenges for PWID in relation to service access and loss of connection and routine. Rapid adaptations to service delivery aiming to help mitigate the risks of COVID-19 infections, were appreciated and some changes such as relaxation of supervised daily OST consumption were viewed positively. However, those facing the greatest barriers to remote service provision at a time of heightened isolation and loss of routine may require tailored, more intensive support.

## Have you obtained ethical approval for the conduct of your study?

Yes

## Data sharing

Interview transcripts are available on request from University of Bristol Research Data Storage Facility.

## Funding sources

This work is funded by the Elizabeth Blackwell Institute Rapid Response COVID-19 scheme and supported by the National Institute for Health Research, Applied Research Collaboration West (NIHR ARC West) and NIHR Health Protection Research Unit (HPRU) in Behavioural Science and Evaluation. The views expressed in this article are those of the authors and not necessarily those of the NIHR, the Department of Health and Social Care, Public Health England, the Wellcome Trust or the Elizabeth Blackwell Institute.

JK and JH are partly funded by National Institute for Health Research Applied Research Collaboration West (NIHR ARC West) and NIHR HPRU in Behavioural Science and Evaluation. MH is funded by NIHR HPRU in Behavioural Science and Evaluation.

LH is funded by the Wellcome Trust (209158/Z/17/Z). AH is a Public Health Specialty Registrar employed by Hampshire Hospitals Foundation Trust. M-JL is funded by the Elizabeth Blackwell Institute, University of Bristol and the Development and Alumni Relations Office, University of Bristol. JS is employed by the University of Bath on a substantive academic contract.

HF is employed by the University of Bristol and her work is funded by BaNES Swindon and Wiltshire NHS Clinical Commissioning Group. She is also employed at the University of Bath on a temporary academic contract.

## Declarations of Interest

None

## Appendix Supplementary materials

Image, application 1

## References

[bib0001] Ali F., Russell C., Nafeh F., Rehm J., LeBlanc S., Elton-Marshall T. (2021). Changes in substance supply and use characteristics among people who use drugs (PWUD) during the COVID-19 global pandemic: A national qualitative assessment in Canada. International Journal of Drug Policy.

[bib0002] Atchison C.J., Bowman L., Vrinten C., Redd R., Pristerà P., Eaton J.W., Ward H. (2020). Perceptions and behavioural responses of the general public during the COVID-19 pandemic: A cross-sectional survey of UK Adults. MedRxiv.

[bib0003] Baker, C., Brown, J., & Barber, S. (2021). *Coronavirus: A history of English lockdown laws*. https://commonslibrary.parliament.uk/research-briefings/cbp-9068/.

[bib0004] Benzano D., Ornell F., Schuch J.B., Pechansky F., Sordi A.O., von Diemen L., Kessler F.H.P. (2021). Clinical vulnerability for severity and mortality by COVID-19 among users of alcohol and other substances. Psychiatry Research.

[bib0005] Black C. (2021).

[bib0006] Braun V., Clarke V. (2013).

[bib0007] Braun V., Clarke V. (2020). One size fits all? What counts as quality practice in (reflexive) thematic analysis?. Qualitative Research in Psychology.

[bib0008] Bursztynsky J. (2019). https://www.cnbc.com/2019/01/15/americans-more-likely-to-die-from-opioid-overdose-than-car-accident.html.

[bib0009] Cabinet Office. (2021). *Coronavirus (COVID-19): Meeting with others safely (social distancing)*. https://www.gov.uk/government/publications/coronavirus-covid-19-meeting-with-others-safely-social-distancing/coronavirus-covid-19-meeting-with-others-safely-social-distancing.

[bib0010] Chiappini S., Guirguis A., John A., Corkery J.M., Schifano F. (2020). COVID-19: The Hidden Impact on Mental Health and Drug Addiction. Frontiers in Psychiatry.

[bib0011] Croxford S., Emanuel E., Ibitoye A., Njoroge J., Edmundson C., Bardsley M., Heinsbroek E., Hope V., Phipps E. (2021). Preliminary indications of the burden of COVID-19 among people who inject drugs in England and Northern Ireland and the impact on access to health and harm reduction services. Public Health.

[bib0012] Department of Health and Social Care. (2021). *COVID-19: Guidance for commissioners and providers of services for people who use drugs and alcohol*. https://www.gov.uk/government/publications/covid-19-guidance-for-commissioners-and-providers-of-services-for-people-who-use-drugs-or-alcohol/covid-19-guidance-for-commissioners-and-providers-of-services-for-people-who-use-drugs-or-alcohol.

[bib0013] Figgatt M.C., Salazar Z., Day E., Vincent L., Dasgupta N. (2021). Take-home dosing experiences among persons receiving methadone maintenance treatment during COVID-19. Journal of Substance Abuse Treatment.

[bib0014] Frank D. (2021). A chance to do it better: Methadone maintenance treatment in the age of Covid-19. Journal of Substance Abuse Treatment.

[bib0015] Genberg B.L., Astemborski J., Piggott D.A., Woodson-Adu T., Kirk G.D., Mehta S.H. (2021). The health and social consequences during the initial period of the COVID-19 pandemic among current and former people who inject drugs: A rapid phone survey in. Drug and Alcohol Dependence.

[bib0016] Gillard S., Dare C., Hardy J., Nyikavaranda P., Olive R.R., Shah P., Birken M., Foye U., Ocloo J., Pearce E., Stefanidou T., Pitman A., Simpson A., Johnson S., Lloyd-Evans B. (2020). Experiences of living with mental health problems during the COVID-19 pandemic in the UK: A coproduced, participatory qualitative interview study. MedRxiv.

[bib0017] Grebely J., Cerdá M., Rhodes T. (2020). COVID-19 and the health of people who use drugs: What is and what could be?. International Journal of Drug Policy.

[bib0018] Hagger M.S., Smith S.R., Keech J.J., Moyers S.A., Hamilton K. (2020). Predicting Social Distancing Intention and Behavior During the COVID-19 Pandemic: An Integrated Social Cognition Model. Annals of Behavioral Medicine.

[bib0019] Hurley E.A., Piña K., Cegielski V., Noel-MacDonnell J.R., Miller M.K. (2021). Recovering from substance use disorders during the early months of the COVID-19 pandemic: A mixed-methods longitudinal study of women in Kansas City. Journal of Substance Abuse Treatment.

[bib0020] Jacka B.P., Phipps E., Marshall B.D.L. (2020). Drug use during a pandemic: Convergent risk of novel coronavirus and invasive bacterial and viral infections among people who use drugs. International Journal of Drug Policy.

[bib0099] John, E., & Butt, A. (2020). Drug-related deaths by local authority, England and Wales [Dataset]. Office for National Statistics. https://www.ons.gov.uk/peoplepopulationandcommunity/birthsdeathsandmarriages/deaths/datasets/drugmisusedeathsbylocalauthority.

[bib0021] Maxwell J.A. (2012).

[bib0022] Ministry of Housing, Communities and Local Government. (2020). *£3.2 million emergency support for rough sleepers during coronavirus outbreak*. https://www.gov.uk/government/news/3-2-million-emergency-support-for-rough-sleepers-during-coronavirus-outbreak.

[bib0023] Platt L., Minozzi S., Reed J., Vickerman P., Hagan H., French C., Jordan A., Degenhardt L., Hope V., Hutchinson S., Maher L., Palmateer N., Taylor A., Bruneau J., Hickman M. (2017). Needle syringe programmes and opioid substitution therapy for preventing hepatitis C transmission in people who inject drugs. Cochrane Database of Systematic Reviews.

[bib0024] Public Health England (2020).

[bib0025] Public Health England, Public Health Scotland, Public Health Wales, & Public Health Agency Northern Ireland (2020).

[bib0026] Rodda L.N., West K.L., LeSaint K.T. (2020). Opioid Overdose-Related Emergency Department Visits and Accidental Deaths during the COVID-19 Pandemic. Journal of Urban Health-Bulletin of the New York Academy of Medicine.

[bib0027] Roe L., Proudfoot J., Tay Wee Teck J., Irvine R.D.G., Frankland S., Baldacchino A.M. (2021). Isolation, Solitude and Social Distancing for People Who Use Drugs: An Ethnographic Perspective. Frontiers in Psychiatry.

[bib0028] Russell C., Ali F., Nafeh F., Rehm J., LeBlanc S., Elton-Marshall T. (2021). Identifying the impacts of the COVID-19 pandemic on service access for people who use drugs (PWUD): A national qualitative study. Journal of Substance Abuse Treatment.

[bib0029] Schofield, J., Dumbrell, J., Browne, T., Bancroft, A., Gallip, I., Matheson, C., & Parkes, T. (2021). *The Impacts of COVID-19 on People Who Use Drugs*. https://covid-drugs.stir.ac.uk/.

[bib0030] Scott J., Millar J., Family H., Kesten J., Hines L.A. (2020). What C-OST? Impact of the Covid-19 Pandemic on People who Receive Opioid Substitution Therapy in Rural Areas. Interim Report number.

[bib0031] Seaman A., Leichtling G., Stack E., Gray M., Pope J., Larsen J.E., Leahy J.M., Gelberg L., Korthuis P.T. (2021). Harm Reduction and Adaptations Among PWUD in Rural Oregon During COVID-19. AIDS and Behavior.

[bib0032] The Independent Scientific Advisory Group for Emergencies (2020). https://www.independentsage.org/wp-content/uploads/2020/11/Inequalities-_i_SAGE_FINAL-draft_corrected.pdf.

[bib0033] Tringale R., Subica A.M. (2021). COVID-19 innovations in medication for addiction treatment at a Skid Row syringe exchange. Journal of Substance Abuse Treatment.

[bib0034] UNAIDS. (2020). *COVID-BLOG - Assisting people who use drugs to receive methadone treatment during COVID-19 lockdown in Kazakhstan*. https://www.unaids.org/en/20200424_Kazakhstan_ost.

[bib0035] Vasylyeva T., Smyrnov P., Strathdee S., Friedman S.R. (2020). Challenges posed by COVID-19 to people who inject drugs and lessons from other outbreaks. Journal of the International Aids Society.

[bib0036] Whitfield M., Reed H., Webster J., Hope V. (2020). The impact of COVID-19 restrictions on needle and syringe programme provision and coverage in England. The International Journal on Drug Policy.

[bib0037] Wilkinson R., Hines L., Holland A., Mandal S., Phipps E. (2020). Rapid evidence review of harm reduction interventions and messaging for people who inject drugs during pandemic events: Implications for the ongoing COVID-19 response. Harm Reduction Journal.

[bib0038] Wisse E., Burke-Shyne N., Chang J., Southwell M. (2021). COVID-19 and people who use drugs; seizing opportunity in times of chaos. The International Journal on Drug Policy.

[bib0039] Zolopa C., Hoj S., Bruneau J., Meeson J.-S., Minoyan N., Raynault M.-F., Makarenko I., Larney S. (2021). A rapid review of the impacts of “Big Events ” on risks, harms, and service delivery among people who use drugs: Implications for responding to COVID-19. International Journal of Drug Policy.

[bib0040] Zvolensky M.J., Garey L., Rogers A.H., Schmidt N.B., Vujanovic A.A., Storch E.A., Buckner J.D., Paulus D.J., Alfano C., Smits J.A.J., O'Cleirigh C (2020). Psychological, addictive, and health behavior implications of the COVID-19 pandemic. Behaviour Research and Therapy.

