# Supplementary material for: Living Under Coronavirus and Injecting Drugs in Bristol (LUCID-B): A qualitative study of experiences of COVID-19 among people who inject drugs
Source: Int J Drug Policy. 2021 Dec;98:103391. doi: 10.1016/j.drugpo.2021.103391 (PMC8289673; doi:10.1016/j.drugpo.2021.103391)
Supplement: Supplementary file 1 [file mmc1.docx]

**Supplementary materials. Interview topic guide**

1. ***Introduction and audio consent***

- Thanks, introduce self.
- Check in safe and confidential space.
- Re-state purpose of the interview. We are working with BDP to find out how the COVID-19 pandemic and social distancing measures are affecting people who inject drugs. This information will be used to help improve the way services, like BDP, for people who inject drugs are provided during the pandemic.
- Consent – explain voluntary participation, right to withdraw and to not answer questions, audio recording, anonymous quotes
- Check for questions
- Switch audio recorder on - For the audio recording, can I check that:
- You read and understood the study information sheet?
- You understand that all information will be kept confidential unless you indicate that you are going to harm yourself or someone else? Only in this situation will I pass on information to BDP.
- You know that taking part in the interview is voluntary and you are free to stop the interview at any point?
- You agree to our conversation being audio recorded?
- You understand that quotations from the interview may be used to illustrate our findings, but it will not be possible to trace who said them?
- You understand that the anonymous data from the study, including a typed copy of your interview with identifying details removed, may be seen and used by other researchers, for ethically approved research projects, on the understanding that confidentiality will be maintained.
- You understand that authorised and responsible individuals may look at sections of the study data, to carry out monitoring for the research sponsor – the University of Bristol.
- You agree to take part in the study?

**Unique identifier**

If you complete the interview but change your mind afterwards, you can withdraw from the study until a week afterwards by contacting Lindsey Hines. Her details are on the information sheet. To enable us to find your interview file please can you tell me your initials and month/year of your birth? You will need to provide this information if you contact us to withdraw from the study.

***START NEW AUDIO RECORDING***

**Begin recording by stating the initials and month/year of birth.**

1. ***Background questions***

How old are you?

How would you describe your current housing status? Was your housing situation different pre-lockdown?

Are you currently injecting drugs?

If yes:

How long have you been injecting drugs for?

Which substances are you injecting at the moment?

Are you using any other substances at the moment?

If no:

When did you last inject?

How long have you been injecting drugs for prior to this?

Are you scripted? If so, what are you using (methadone, buprenorphine [Subutex]) Were you on a script before the lockdown?

1. ***Response to COVID-19***

In March, strict measures were placed on public life to limit the spread of the coronavirus. Initially the guidance was for people to only leave home to exercise once a day, travel to and from work when it is "absolutely necessary", shop for essential items and fulfil any medical or care needs. I’ll call these measures ‘lockdown’. The guidance also recommended people stay at least 2 metres from each other – I’ll call this ‘social distancing’. **This guidance has now been updated. As of Saturday 4^th^ July, pubs, restaurants and hairdressers were able to reopen and two households are able to meet up in any setting with social distancing measures. Where it is not possible to stay 2 metres apart, guidance allows people to keep a social distance of ‘one metre plus’ which means staying one metre apart with other measures to reduce the risk like wearing a face mask or turning away from the person.**

**Are you doing anything to avoid getting coronavirus? If so, what? If not, for what reason?**

PROMPTS

| - Hand hygiene | - Keeping using spaces clean |
| --- | --- |
| - Following good respiratory hygiene (covering mouth when coughing) | - **Practicing physical distancing – Staying 2 metres away from people not living and/or socially isolating with** |
| - Not carrying drugs in mouth, rectum or vagina | - Self-Isolation: If have symptoms or live with someone who has been diagnosed or has symptoms, staying home for 14 days |
| - Not sharing drug using paraphernalia, particularly smoking, snorting and injecting equipment |  |

**What has made doing these things easier / more difficult?**

**When/where is it easier to follow these measures? (Buying drugs – are dealers wearing masks etc., any social distancing practices used, using drugs, at home, at work)**

**What could be done to help you follow the social/physical distancing advice?** (e.g. sources of information/ advice)

**Have you experienced any COVID-19 symptoms (e.g. high temperature, new and continuous cough)? If yes, did you access healthcare services? Easy? Difficult? Tested?**

**How concerned are you about coronavirus? How does coronavirus compare to other priorities you have or challenges you’re facing at the moment?**

**Have you received any information about how to protect yourself from coronavirus?**

1. ***Impact of COVID-19***

**What has been the biggest challenge of the social distancing / lockdown measures due to coronavirus for you?**

**How has this changed over the course of the lockdown?**

**How have the social distancing / lockdown measures due to coronavirus affected your drug use? Negative effects? Positive effects?**

PROMPTS (if needed)

| Patterns of drug use   - Where, who with, how, when, what, how often - Amount bought (stockpiling) / used | Drugs effects   - Strength / unexpected suggesting potential adulteration - Overdose experience (personal and others) - Withdrawals |
| --- | --- |
| Harm reduction practices   - **Injecting site (conditions of and changes in site)** - Sharing/re-using/cleaning equipment - Wearing gloves/face mask | Accessing injecting equipment / harm reduction services   - **Source/location of service – know of pharmacies stopping NSP?** - **Receipt of harm reduction advice – written / verbal** - Frequency and amount collected (stocking up) - **Offered Naloxone** |
| Opioid substitution therapy (OST)   - Returning to - Signing up through homeless healthcare team (CHART workers) - Increasing dose - Pharmacy collection, homeless health service collection, home delivery, peer sharing | Access to healthcare services   - Source - How easy/difficult |
| - OST collected weekly vs daily - Shared care appointment by telephone: What have you felt about having telephone rather than face to face contact with shared care worker? | Drug related health issues   - Overdose - Skin and soft tissue infections - Abscesses - **Testing/treatment for viruses (Hep C, HIV etc.)** |
| Psychological stress (isolation, anxiety, boredom, activities to occupy time, virtual communication / use of internet) | Changes to income / how people are coping financially   - Benefits - Ability to pay for drugs (e.g. setting up tab) |

**What are the reasons for the changes in your drug use?**

PROMPTS (if needed)

| Supply of drug | Harm reduction advice |
| --- | --- |
| Source of income | Social / physical distancing advice |
| Housing (isolating at home, hotels, street homeless) | Desire to reduce drug use |
| Access to needle exchange and injecting equipment | Contact with service providers e.g. shared care workers reduced face-to-face contact |
| Crowded pharmacies at beginning of lockdown | Psychological stress (isolation, anxiety,boredom) |
|  |  |

**What things could help overcome the challenges of coronavirus? E.g. support from services or any other changes which would help you at the moment?**

1. ***Changes to services***

**Have you noticed any changes to drug services during coronavirus? Examples? How did you find out about these changes (e.g. word of mouth, posters, written information in pharmacy packs)?**

Have services or support you’ve previously engaged with stopped or changed? How has this affected you? Negative? Positive? Changes to online, telephone communication rather than face to face?

**What changes to drug services have helped/not helped you during coronavirus?**

[IF NOT MENTIONED IN RESPONSE TO THE ABOVE] BDP has changed its approach due to coronavirus to meet the needs of people who inject drugs:

- Central needle exchange (Brunswick Square) remains open as a doorstep service- can now go inside the building one at a time. Offered Naloxone.
- Mobile needle exchange comprising foot outreach to all hotels/hostels and areas of the city where people who inject drugs meet
- Harm reduction van – since beginning of lockdown no longer going to Hartcliffe and Knowle (previously visited 3 times per week), moved to Travel Lodge to reach clients in hotels and now goes to Bedminster where has high use.
- Home delivery service available to all, set up by phone, particularly to areas beyond the city centre – barriers to using local pharmacy NSP for people accessing this service?
- OST delivery service to those isolating or shielding where friends/family or pharmacists are unable to help.
- **A nurse accompanies outreach walks and visits to hotel and hotels providing a range of services including wound care.**
- BDP / DHI groups – PRISM (LGBT) runs over Zoom, drug treatment groups run one-to-one by phone, Mutual aid (AA, NA, SMART recovery by Zoom).
- No community detox for opiates. ACER unit closed

**Have you experienced any of these changes?**

**If yes:**

- **What’s done well/not so well? Receipt of harm reduction advice, length/confidentiality of conversations with workers**
- **What could be done to improve these services?**

**If no:**

- **What are barriers to using these services?**
- **What could be done to improve these services?**

**What additional services or adaptations would be useful?**

**What do you think should happen after lockdown measures are lifted? For example would you keep any of the service changes (e.g. OST prescription pick-ups)?**

**Close**

That is the end of my questions. Is there anything we have not discussed about your experience of coronavirus or lockdown that you would like to add?

End of audio recording

Thanks for participating

Remind that can withdraw from the study until a week afterwards by contacting Lindsey Hines (details on information sheet).

Explain how to access £10 as a thank you for their time.

In the event that a participant becomes upset during the interview:-

1. Remind the participant that they can terminate the interview at any time.
2. Offer contact details of local services:
   1. **Bristol Samaritans** (116 123 free from any phone or 0330 094 5717 local call charges apply, 37 St. Nicholas Street, Bristol BS1 1TP) provides confidential emotional support to those in need of a safe space to talk over problems, feelings, stress or anything that maybe worrying them.
   2. **The Sanctuary Bristol** (Mobile: 07709 295 661 or office number: 0117 9542952, Email address:awp.bmhsanctuary@nhs.net) currently running a telephone only service 7 days a week from 4-10pm. After an initial informal assessment they will allocate a time slot for people during the evening when staff will call back and offer support.

Other unmet needs relating to drug use (e.g. injecting equipment or advice) refer the participant to BDP for further support (telephone number 0117 987 6000)
